# Stem Cells and Platelet-Rich Plasma Enhance the Healing Process of Tendinitis in Mice

**DOI:** 10.1155/2019/1497898

**Published:** 2019-06-02

**Authors:** Rosangela Alquieri Fedato, Júlio César Francisco, Gabriel Sliva, Lúcia de Noronha, Márcia Olandoski, Jose Rocha Faria Neto, Priscila Elias Ferreira, Rossana Baggio Simeoni, Eltyeb Abdelwahid, Katherine Athayde Teixeira de Carvalho, Luiz César Guarita-Souza

**Affiliations:** ^1^Experimental Laboratory of Institute of Biological and Health Sciences of Pontifical Catholic University of Paraná (PUCPR), Rua Imaculada Conceição 1155, 80215-901 Curitiba, PR, Brazil; ^2^Positivo University (UP), Rua Professor Pedro Viriato Parigot de Souza, 5300, 80710-570 Curitiba, PR, Brazil; ^3^Cell Therapy and Biotechnology in Regenerative Medicine Research Group, Pelé Pequeno Príncipe Institute, Avenida Silva Jardim 1632, 80250-200 Curitiba, PR, Brazil; ^4^Feinberg School of Medicine, Feinberg Cardiovascular Research Institute, Northwestern University, 303 E. Chicago Ave., Chicago, IL 60611, USA

## Abstract

**Objective:**

Achilles tendon pathologies occur frequently and have a significant socioeconomic impact. Currently, there is no evidence on the best treatment for these pathologies. Cell therapy has been studied in several animal models, and encouraging results have been observed with respect to tissue regeneration. This study is aimed at evaluating the functional and histological effects of bone marrow stem cell or platelet-rich plasma implantation compared to eccentric training in the treatment of Achilles tendinopathy in rats.

**Methods:**

Fourty-one male Wistar rats received collagenase injections into their bilateral Achilles tendons (collagenase-induced tendinopathy model). The rats were randomly divided into four groups: stem cells (SC), platelet-rich plasma (PRP), stem cells+platelet-rich plasma (SC+PRP), and control (eccentric training (ET)). After 4 weeks, the Achilles tendons were excised and subjected to biomechanical and histological analyses (Sirius red and hematoxylin-eosin staining).

**Results:**

Biomechanical assessments revealed no differences among the groups in ultimate tensile strength or yield strength of the tendons (*p* = 0.157), but there were significant differences in the elastic modulus (MPa; *p* = 0.044) and maximum tensile deformation (*p* = 0.005). The PRP group showed the greatest maximum deformation, and the SC group showed the highest Young's modulus (elasticity) measurement. In histological analysis (hematoxylin-eosin and Sirius red staining), there were no differences among the groups.

**Conclusion:**

PRP and SC+PRP yielded better biomechanical results than eccentric training, showing that these treatments offer better tend function outcomes. This theoretical rationale for the belief that cell therapies can serve as viable alternatives to current treatments chronic fibrotic opens the door for opportunities to continue this research.

## 1. Introduction

Tendon disorders are common in clinical practice and can cause significant morbidity, pain, and consequent practical reductions in both work and physical activity [1]. Because tendons are poorly vascularized [2], their healing potential is poor if they are damaged by acute or chronic lesions [3]. Work overload can cause them to undergo fibrosis-related structural changes. This situation predisposes patients to chronic pain and tendon rupture [4].

Achilles tendon pathologies are especially common and tend to be symptomatic [4]. Their prevalence varies and depends on the type and intensity of specific sports activities. The prevalence of Achilles tendinopathies can reach 66% among runners [5].

The use of platelet-rich plasma (PRP) as a treatment for tendinitis promotes cell proliferation and chemotaxis. PRP also enhances the healing potential by stimulating neovascularization and activating growth factors that increase gene transcription and protein synthesis. These changes trigger cell proliferation and cell differentiation, resulting in faster and more effective healing [6].

Nonetheless, in orthopedics, the results have not been so encouraging. Studies on the isolated administration of growth factors have shown more favorable results than studies on PRP administration [6]. However, there are still no randomized controlled trials to prove this notion [7].

As for bone marrow mononuclear stem cells, their ability to differentiate into multiple conjunctival cell types, including chondrocytes [8, 9] and tenocytes [10, 11], has already been well characterized in preclinical studies. Moreover, it is well known that stem cells (SCs) can induce the formation of linear arrangements of type I collagen [12] which increases the elastic modulus, yield strength, and resistance to deformation and improves the biomechanical characteristics of the tendon [13, 14].

SCs have been used experimentally for the treatment of superficial flexor tendinitis in horse toes, which resulted in significantly lower recurrence rates than those in horse toes that were subjected to standard treatments [15–17]. Nevertheless, these results are not persistent, because 4 months after injury, the recurrence rates are similar between the two groups [15].

In the present experimental rat study, our objective was to verify the functional and histological effects of injecting SC, PRP, or SC+PRP compared with eccentric training (ET) protocols in treating Achilles tendinitis induced by type I collagenase.

## 2. Materials and Methods

This was an experimental study performed according to the guidelines of the Brazilian College of Animal Experimentation (COBEA) and approved by the Ethics Committee (Pontifical Catholic University of Paraná (CEUA PUCPR), under ID number 01037). To assess these effects of the SC, PRR, SC+PRP, and ET protocols, we performed a histological analysis using hematoxylin-eosin and Sirius red staining. Biomechanical evaluations were also performed 4 weeks after treatment on the 41 Wistar male rats weighing between 250 and 350 g used in the study. All animals were subjected to bilateral percutaneous applications of type IA collagenase in the Achilles tendon regions, by clinical palpation with the ankle flexed at 90°, at doses of 250 IU (30 *μ*l) and concentrations of 10 mg/ml [16], dissolved in 0.09% saline solution for a volume of 0.5 ml, after the intraperitoneal (IP) administration of a combined anesthesia regimen of 5% ketamine (80 mg/kg) and 2% xylazine hydrochloride (10 mg/kg). Five days after the injuries, the animals were randomized into 4 groups: SC group (*n* = 10), PRP group (*n* = 10), SC+PRP group (*n* = 12) (the rats were reanesthetized to administer SCs or PRP in groups SC, PRP, and SC+PRP), and an eccentric exercise protocol group (ET, control group, *n* = 9).

SCs were obtained by collecting ~2 ml of blood from the rats' iliac crests. The blood was then separated by Ficoll density gradient centrifugation using IMDI culture medium (Iscove's modified Dulbecco's medium) supplemented with antibiotics (1% penicillin and streptomycin), according to the technique described by Boyum [17]. The SC specimens were resuspended and applied alone percutaneously to the animals' bilateral Achilles tendons.

PRP was prepared by collecting ~1-2 ml of blood via cardiac puncture according to Anitua's technique. This was followed by serial homogenization and centrifugation [18]. Similarly, the specimens obtained from each animal for the PRP preparations were reapplied alone percutaneously to the Achilles tendons after resuspension.

**Figure 1:**
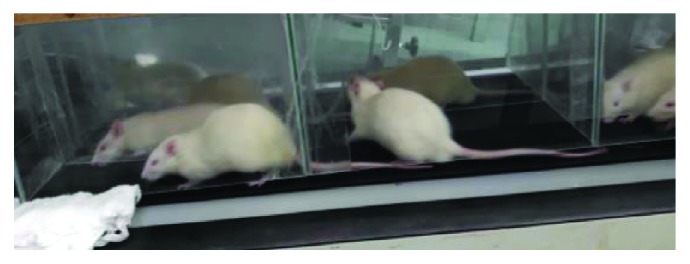
Animals performing eccentric training.

For animals in the SC+PRP group, the two steps described above were carried out concomitantly. The animals in the ET group started an exercise training protocol on individual 15° inclination treadmills (Figure 1) following an adaptation regimen as follows: 1 km/h for 15 minutes in the first 2 weeks, gradually increasing to 45 minutes, followed by 17 m/min (1 km/h) for 1 hour three times a week for 2 additional weeks [19].

The animals were administered a subcutaneous dose of carprofen analgesic at 5 mg/kg per day via abdominal application for 72 hours after the implant procedure. The animals were killed 4 weeks later to comparatively analyze the groups. One tendon was analyzed histologically, and the contralateral tendon was subjected to biomechanical testing.

### 2.1. Functional Analysis

The biomechanical evaluations were performed on an EMIC DL500 device, which was used to assess the parameters of ultimate tensile strength, yield strength, maximum deformation, and elastic modulus.

### 2.2. Histology

For the histological evaluation, the Achilles tendons were mounted as 5 *μ*m thick sections on histological slides and stained with hematoxylin and eosin to characterize the cells and extracellular matrix. Cellularity, vessels, and collagen fibers were scored from 1 (normal) to 4 (significant changes). The criteria to evaluate the tendons included the following: tenocyte morphology and density; presence of hemorrhage, neovascularization, and inflammatory infiltrates; linearity and undulation of collagen fibers; and epitendon thickness (according to previous publications on histological evaluations of tendinopathy, as described by Urdzikova et al.) [20].

To evaluate the proportions of type I and III collagens in the affected tissue, the slides were stained with Sirius red, and a computerized analysis was carried out to assess the percentages. This approach, as reported by Nixon et al., has been used in other studies to evaluate tendinopathy [21].

### 2.3. Statistical Analysis

Quantitative variables were compared using the analysis of variance (ANOVA) or the Kruskal–Wallis test, whereas normality was assessed using the Shapiro–Wilk test. For qualitative variables, a Fisher or Chi-square test was conducted. Any *p* value < 0.05 indicated a statistical significance (data were analyzed using IBM SPSS Statistics software, v.20.0; IBM Corp., Armonk, NY, USA).

## 3. Results

Four weeks after treatment, we obtained the following specimens for the histological analyses:

- 8 tendons each from the SC and PRP groups
- 6 tendons from the SC+PRP group
- 9 tendons from the ET (control) group

For the biomechanical analyses, we obtained the following specimens:

- 10 tendons each from the SC and PRP groups
- 12 tendons from the SC+PRP group
- 9 tendons from the ET (control) group

### 3.1. Functional Analysis

Regarding biomechanical testing, descriptive statistics of each parameter are presented in Table 1.

**Figure 2:**
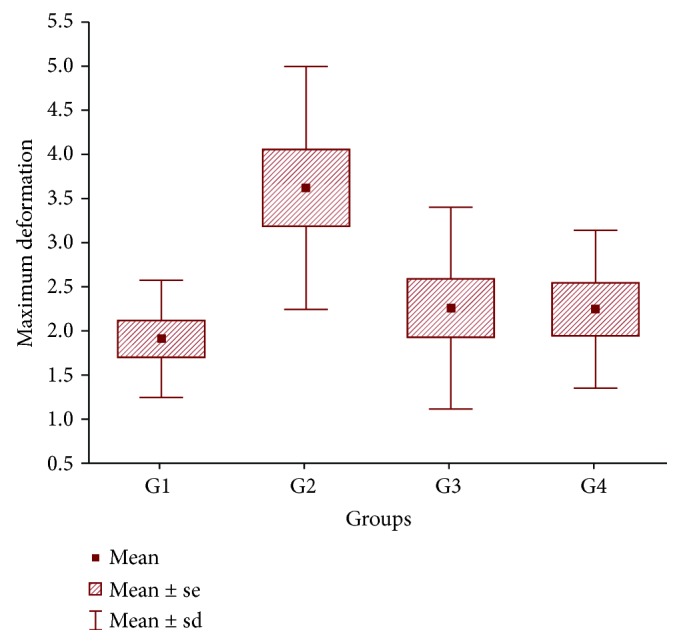
A histogram showing maximum deformation, which is significantly higher in the PRP group. G1 = SC; G2 = PRP; G3 = SC+PRP; G4 = ET. PRP: platelet-rich plasma; SC: stem cell: sd: standard deviation; se: standard error.

**Figure 3:**
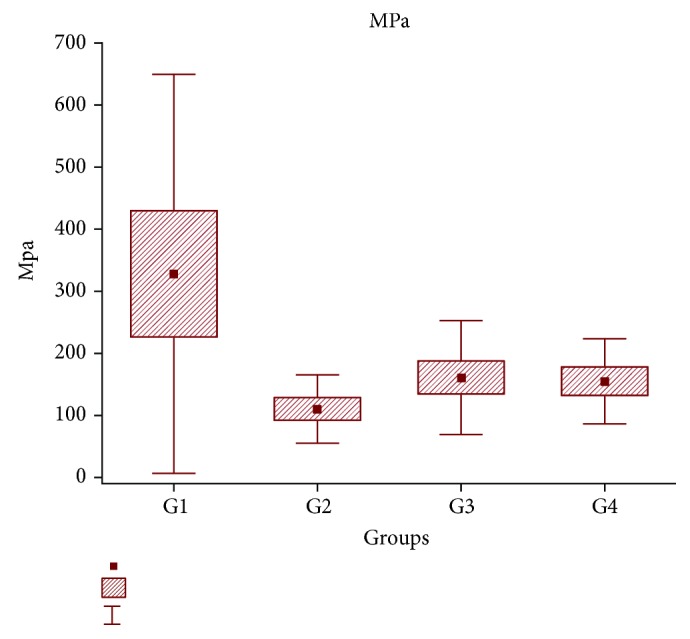
A histogram showing the elastic modulus, which is significantly lower in the PRP group and higher in the SC group. G1 = SC; G2 = PRP; G3 = SC+PRP; G4 = ET. MPa: elastic modulus; SC: stem cell; PRP: platelet-rich plasma; sd: standard deviation; se: standard error.

Regarding ultimate tensile strength and yield strength, there were no statistically significant differences among the groups (*p* = 0.157). However, differences were observed in the maximum deformation (Figure 2) and elastic modulus (Figure 3) (*p* = 0.005 and *p* = 0.044, respectively) when the groups were compared pairwise (post hoc least significant difference (LSD) test, *p* < 0.05).  Table 2 below shows the *p* values for these comparisons.

The PRP group had significantly better results for maximum deformation and elastic modulus than all the other groups. The SC group had an elastic modulus result that was significantly higher than that of the other groups (Figures 2 and 3).

### 3.2. Histological Analysis

The tendons were prepared by staining the tissue sections with hematoxylin and eosin. Next, they were evaluated on a scale of 1 to 4 (where 1 = normal and 4 = highly abnormal) according to the following parameters: morphology and density of tenocytes, presence of hemorrhage, neovascularization, inflammatory cell infiltrate, linearity and undulation of collagen fibers, and epithelial thickness. Using this scoring system, each tissue section was scored between 8 (normal) and 32 (maximum abnormality), and the results were used in the statistical analysis.

**Figure 4:**
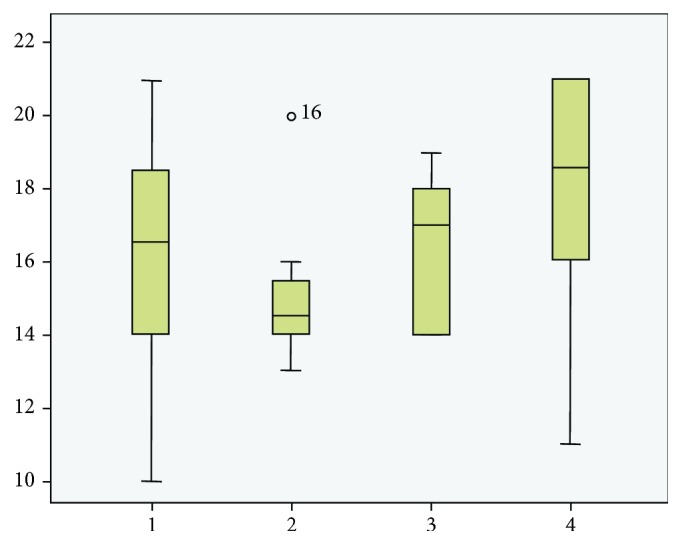
A histogram showing the histological scores in each group. 1 = SC; 2 = PRP; 3 = SC+PRP; 4 = ET. SC: stem cell; PRP: platelet-rich plasma; ET: eccentric training.

After analyzing the score results, there were no statistically significant differences in histological scores among the groups (*p* = 0.133; Kruskal–Wallis test). The scores varied from 10 to 21 in the SC group (mean ± SD: 16.1 ± 3.5), from 13 to 20 in the PRP group (15.1 ± 2.1), from 14 to 19 in the SC+PRP group (16.5 ± 2.07), and from 11 to 21 in the control (ET) group (18.5 ± 3.8) (Figure 4).

Bleeding was not observed in any of the tissue sections in any group. All tissue sections in all groups received a score of 2 (slight increase) for neovascularization, with the exception of one tissue section belonging to the SC group, which received a score of 1 (none) (Table 3).

The inflammatory cell infiltrates received score 1 (not present) or 2 (slight increase) among groups, as illustrated in Table 4.

Regarding the linearity of the collagen fibers, the scores also varied only in 1 (normal) or 2 (more than 50% of linear collagen fibers) among groups (Table 5).

Undulation of the collagen fibers varied from normal (score value 1: all fibers are undulated) to moderately abnormal (score value 3: without undulation) among the groups (Table 6).

**Figure 5:**
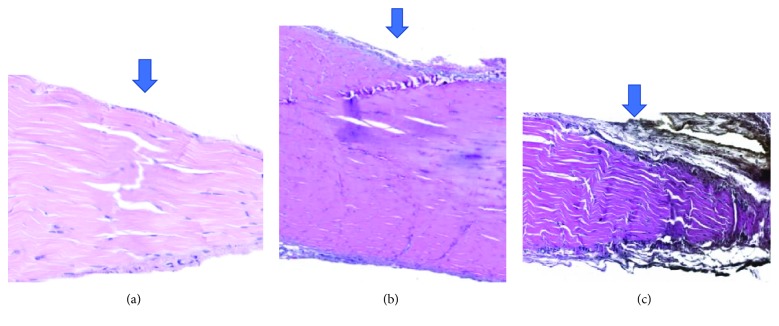
Enlarged images of tissue sections showing an eosin-stained epitendon in one cell layer (normal) or several cell layers (massive fibrosis).

The thickness of the epitendons (Figures 5(a)–5(c)) also showed no significant differences among the groups; however, no group exhibited evidence of normal epitendon organization. Two tissue sections showed maximum abnormalities in the SC group, as presented in Table 7.

**Figure 6:**
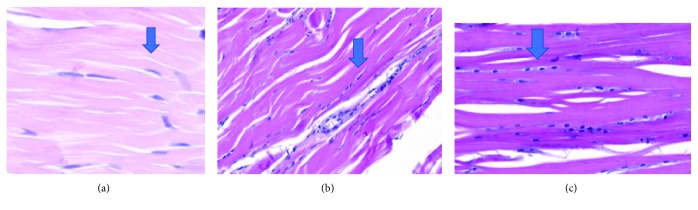
Enlarged images showing variations in the tenocytes, from linear and sparse to rounded and significantly increased.

There were no significant differences in tenocyte morphologies among the groups (*p* = 0.595), as described in Table 8 and Figures 6(a)–6(c).

There was a tendency toward histological normality of the tenocyte densities in groups SC, PRP, and SC+PRP compared with that in the control group (ET) (Kruskal–Wallis test, *p* = 0.073 and *p* = 0.411, respectively). In groups SC, PRP, and SC+PRP, the tissue sections scored 2 or 3 for this parameter, which meant a mild or moderate increase in tenocyte density, respectively. In the control group, this parameter scored 4 (tenocyte layers) (Table 9).

To assess the percentages of type I and III collagens in each group, the tissue sections were stained with Sirius red and analyzed using a software program that calculated the percentage of each collagen in the tissue sections.

**Figure 7:**
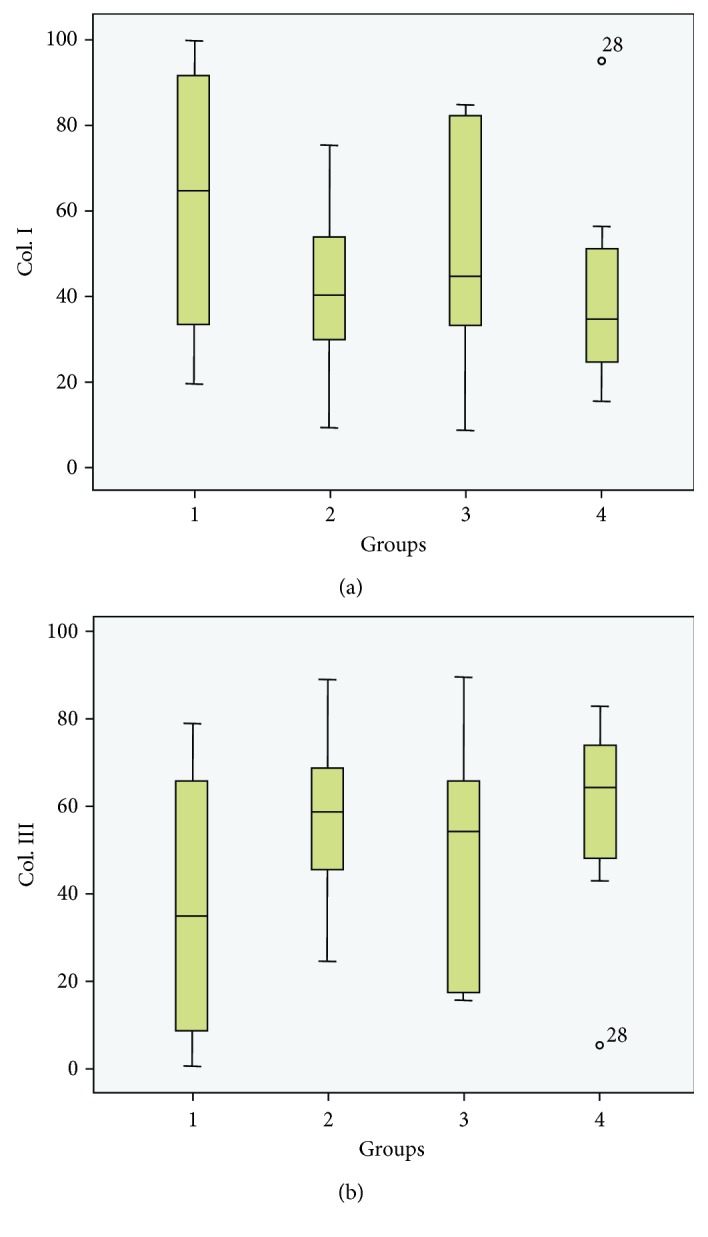
Histograms of the proportions of collagen (types I or III) in each group.

The mean percentage of type I collagen in the SC group was 61.82, and the median value was 64.24; in the PRP group, the mean was 41.18 and the median was 39.71; in the SC+PRP group, the mean was 49.29 and the median was 44.28; in the ET group, the mean was 40.09 and the median was 34.25, as shown in Table 10 and Figure 7(a).

Regarding type III collagen, the mean percentage in the SC group was 38.17 and the median value was 35.76; in the PRP group, the mean was 58.81 and the median was 60.28; in the S+PRP group, the mean was 50.70 and the median was 55.72; in the control group (ET), the mean was 59.90 and the median was 65.74, as demonstrated in Table 11 and Figure 7(b).

There were no significant differences in the percentages of type I and III collagens among the groups (Kruskal–Wallis test, *p* = 0.489) (Figures 6(a)–6(c)).

## 4. Discussion

To date, several studies have compared various conservative modalities used to treat Achilles tendinitis. However, the results are conflicting, indicating that nonhormonal and hormonal anti-inflammatory treatments might have efficacies similar to placebo. Hormonal anti-inflammatories are associated with Achilles tendon rupture when applied to peritendinous regions [8].

Likewise, sclerosing agents such as polidocanol act to prevent neovascularization and alleviate pain, but they are also associated with higher rates of tendon rupture [8]. Unfortunately, surgical treatments are associated with high failure rates [8].

The aforementioned reasons coupled with socioeconomic factors, especially with respect to injuries in elite athletes, have made Achilles tendinitis the target of several animal studies conducted to identify newer, more effective, and safer therapies.

Our data revealed that cell therapy, especially in the groups that received PRP and the SC+PRP combination, was associated with maximum deformation in biomechanical testing. The difference was significantly greater in these two groups than that in the other groups. This parameter is extremely important in the evaluation of Achilles tendinitis because the deformation capacity of tendons (elastic property) forms the basis for proper physiological function. These findings are consistent with other research data, such as that of Nixon et al., whose study involved PRP and tenocytes derived from adipocytes that were used in an animal model of tendinitis (also see Shah et al.'s) [21, 22]. Both studies revealed that the use of growth factors derived from PRP improved the biomechanical characteristics of injured tendons. Our findings corroborate the notion that growth factors derived from PRP are capable of stimulating new tissue formation in these pathological conditions [2, 21, 22].

Our study did not show a statistically significant difference among the groups in the histological evaluations. In contrast to other existing studies, our study compared the groups treated with cell therapies to a group treated with a physiotherapeutic modality not to an injured group that received no treatment. This approach might explain the smaller histological differences observed among our groups. In addition, the absence of histological and biomechanical differences among the groups has been demonstrated in the literature. Shah et al. revealed better biomechanical performances even in the absence of histological differences [22].

This phenomenon was also explained by Zhang et al. They reported a reduction in the expression of the *COX-1* and *COX-2* genes and lower levels of prostaglandins (e.g., PGE2) when rats were treated with PRP in a tendinitis model. In that study, they proved the anti-inflammatory effect of PRP.

The absence of histological and immunohistochemical differences was also mentioned by Parafioriti et al. [23]. In their surgical model of Achilles lesions in rats, although there were significant histological differences 1 week after treatment, they did not persist at 2, 4, and 6 weeks after treatment [23].

In contrast, our data showed a trend toward significant changes in histological tenocyte density in the cell therapy groups. In similar studies with larger sample sizes, the best organization of the extracellular matrix in stem cells groups has already been proven [20].

In addition to obtaining similar results, Crovace et al. showed that SC implantation restored the proportion of type I and III collagens compared to that in controls, thereby proving that tissue regeneration occurs after collagenase-induced tendon injuries in sheep [12].

In accordance with the literature, our data suggest that cell therapies may be more effective than currently available treatments. We have shown a higher efficacy of PRP in the treatment of tendons based on biomechanical test results and tendency toward histological normality of the tenocytes in groups that received cell therapies (SCs and/or PRP). This finding strengthens the notion that factors promoting cell differentiation can reactivate relatively inert cells such as tenocytes and assist in managing pathologies with predominantly fibrotic characteristics. A statistical extrapolation of the data obtained in this study indicates that a larger sample size would likely achieve higher levels of significance.

Despite the animal models indicating a superiority of cell therapy models in the treatment of tendinitis, a recent systematic review reported that randomized clinical trials are necessary for validation. In humans, relevant studies are currently limited to case series, which do not provide strong evidence for direct clinical application and point the need for clinical studies. Nevertheless, cell therapies, which promote medical tourism, are already available in some countries [24, 25].

Our findings support the importance of follow-up research and the need to improve this experimental model. Altogether, these findings offer much hope for effective and lasting treatment of these common and disabling pathologies.

This study is limited by the fact that it is an animal model. Additionally, there is no current standard treatment for Achilles tendinopathy to be used for comparison with potentially new therapeutic modalities.

## 5. Conclusion

We can conclude that, in a rat model of tendinitis, the functional effect of implanted PRP was stronger than that of SC alone, ET, or the SC+PRP combination at 4 weeks after treatment.

Regarding the histological analysis with hematoxylin and eosin alone, there was no statistically significant difference among the groups, either in the total score (sum of the scores of each item analyzed) or in separate analyses of each of the variables including tenocyte morphology and density, presence of hemorrhage, inflammatory cell infiltration, neovascularization, linearity and undulation of collagen fibers, and epithelial thickness.

In the analysis of the percentages of type I and III collagens determined by Sirius red staining, there were no statistically significant differences among the groups at 4 weeks after treatment.

Finally, it was the functional analysis of the biomechanical parameters that revealed a higher efficacy of PRP cell therapy compared to the treatments in the other groups. Tendon samples in the PRP group withstood the greatest deformation when compared to those in the other groups.

Accordingly, there is a theoretical rationale for the belief that cell therapies can serve as viable alternatives to the current treatments for chronic fibrosis disorders. This opens the door for opportunities to continue this research.

## Figures and Tables

**Table 1:** Ultimate tensile strength, yield strength, maximum tensile deformation, and elastic modulus as determined via biomechanical testing.

Variable	Group	*n*	Mean	Median	Minimum	Maximum	Standard deviation	*p* value^∗^
Ultimate tensile strength	SC	10	26.9	27.45	17.8	39.4	7.4	0.157
	PRP	10	24.9	25	16.7	34.4	5.2	
	SC+PRP	12	20.8	22.6	8.7	30.2	5.9	
	ET	9	21.8	22.2	10.8	38.3	8.2	

Yield strength	SC	10	17.9	18.3	11.9	26.3	4.9	0.157
	PRP	10	16.6	16.65	11.2	22.9	3.4	
	SC+PRP	12	13.9	15.1	5.8	20.1	3.9	
	ET	9	14.5	14.8	7.2	25.5	5.5	

Maximum deformation	SC	10	1.9	1.8	1.1	3.1	0.7	0.005
	PRP	10	3.6	3.35	1.8	6	1.4	
	SC+PRP	12	2.3	2.05	0.6	4.1	1.1	
	ET	9	2.2	1.9	1.3	3.8	0.9	

Elastic modulus (MPa)	SC	10	328.1	230.2	83.2	1171.2	321.5	0.044
	PRP	9	155.1	122.3	84.1	278.9	68.6	
	SC+PRP^∗∗^	12	161.1	149.9	14.8	371.4	91.6	
	ET	9	155.1	122.3	84.1	278.9	68.6	

^∗^One-way ANOVA. *p* < 0.05. ^∗∗^An outlier was excluded (equal to -5.14). SC: stem cell; PRP: platelet-rich plasma; ET: eccentric training; ANOVA: analysis of variance.

**Table 2:** Results of pairwise statistical analyses of the differences between the groups with regard to the biomechanical variables.

Compared groups	*p* value^∗^	
	Maximum deformation	MPa
SC vs. PRP	0.001	0.010
SC vs. SC+PRP	0.449	0.031
SC vs. ET	0.498	0.037
PRP vs. SC+PRP	0.005	0.511
PRP vs. ET	0.008	0.587
SC+PRP vs. ET	0.977	0.938

^∗^ *Post hoc* LSD (least significant difference) test; *p* < 0.05. MPa: elastic modulus; SC: stem cell; PRP: platelet-rich plasma; ET: eccentric training.

**Table 3:** Analysis of neovascularization.

Neovascularization	Groups			
	SC	PRP	SC+PRP	ET
1: none	1	0	0	0
	(12.5%)			
2: slight increase	7	8 (100%)	6 (100%)	9 (100%)
	(87.5%)			
Total	8 (100%)	8 (100%)	6 (100%)	9 (100%)

^∗^ *p* value: 0.411 (Kruskal-Wallis test). SC: stem cell; PRP: platelet-rich plasma; ET: eccentric training.

**Table 4:** Analysis of inflammatory cell infiltrates.

Inflammatory cell infiltrates	Groups			
	SC	PRP	SC+PRP	ET
1: none	2 (25%)	1 (12.5%)	0	1 (11.1%)
2: slight increase	6 (75%)	7 (87.5%)	6 (100%)	8 (88.9%)
Total	8 (100%)	8 (100%)	6 (100%)	9 (100%)

SC: stem cell; PRP: platelet-rich plasma; ET: eccentric training.

**Table 5:** Analysis of collagen fiber linearity.

Linearity of collagen fibers	Group			
	SC	PRP	SC+PRP	ET
1: linear (normal)	5 (62.5%)	7 (87.5%)	5 (83.3%)	2 (22.2%)
2: >50% of linear fibers	3 (37.5%)	1 (12.5%)	1 (16.7%)	7 (77.8%)
Total	8 (100%)	8 (100%)	6 (100%)	9 (100%)

SC: stem cell; PRP: platelet-rich plasma; ET: eccentric training.

**Table 6:** Analysis of collagen fiber undulation.

Undulation of the collagen fibers	Groups			
	SC	PRP	SC+PRP	ET
1: undulated	5 (62.5%)	7 (87.5%)	5 (83.3%)	3 (33.3%)
2: slightly undulated	2 (25%)	1 (12.5%)	1 (16.7%)	6 (66.7%)
3: without undulation	1 (12.5%)	0	0	0
Total	8 (100%)	8 (100%)	6 (100%)	9 (100%)

SC: stem cell; PRP: platelet-rich plasma; ET: eccentric training.

**Table 7:** Analysis of epitendon thicknesses.

Epitendon thicknesses	Groups			
	SC	PRP	SC+PRP	ET
2: 3 to 6 cells	4 (50%)	6 (75%)	6 (100%)	4 (44.4%)
3: 7 to 15 cells	2 (25%)	2 (25%)	0	5 (55.6%)
4: Massive fibrosis	2 (25%)	0	0	0
Total	8 (100%)	8 (100%)	6 (100%)	9 (100%)

SC: stem cell; PRP: platelet-rich plasma; ET: eccentric training.

**Table 8:** Analysis of tenocyte morphology.

	Groups			
Tenocyte morphologies	SC	PRP	SC+PRP	ET
1: linear	2	0	0	1 11.1%
	25%			
2: slightly oval	2	5 62.5%	2 33.3%	2 22.2%
	25%			
3: moderately rounded	3	3 37.5%	3	5 55.6%
	37.5%		50%	
4: predominantly rounded	1	0	1 16.7%	1 11.1%
	12.5%			
Total	8	8	6	9

*p* value: 0.595 (Chi-square test). SC: stem cell; PRP: platelet-rich plasma; ET: eccentric training.

**Table 9:** Analysis of tenocyte density.

Tenocytes density	Groups			
	SC	PRP	SC+PRP	ET
1: sparse (normal)	1 12.5%	0	0	1 11.1%
2: mild increase	5 62.5%	6 75%	2 33.3%	2 22.2%
3: moderate increase	2 25%	2 25%	4 66.7%	0
4: cell layers	0	0	0	6 66.7%
Total	8	8	6	9

*p* value: 0.073 (Chi-square test), SC: stem cell; PRP: platelet-rich plasma, ET: eccentric training.

**Table 10:** Analysis of the percentage of type I collagen in each group.

	Type I collagen (%)			
	Group			
	SC	PRP	SC+PRP	ET
*n*	8	8	6	9
Mean	61.82	41.18	49.29	40.09
Median	64.24	39.71	44.28	34.25
Minimum	34.32	23.94	17.73	21.32
Maximum	89.33	58.43	80.85	58.86
Standard deviation	11.63	7.29	12.27	8.13
*p* value^∗^				0.489

**Table 11:** Analysis of the percentage of type III collagen in each group.

	Type III collagen (%)			
	Groups			
	SC	PRP	SC+PRP	ET
*N*	8	8	6	9
Mean	38.17	58.81	50.70	59.90
Median	35.76	60.28	55.72	65.74
Minimum	10.66	41.56	19.14	41.13
Maximum	65.68	76.05	82.26	78.67
Standard deviation	11.63	7.29	12.27	8.14
*p* value^∗^				0.489

^∗^Nonparametric Kruskal–Wallis test. SC: stem cell; PRP: platelet-rich plasma; ET: eccentric training.

## Data Availability

The data used to support the findings of this study are available from the corresponding author upon request.
